# Inverse Association between Metformin and Amiodarone-Associated Extracardiac Adverse Events

**DOI:** 10.7150/ijms.39342

**Published:** 2020-01-16

**Authors:** Sayoko Kinoshita, Kouichi Hosomi, Satoshi Yokoyama, Mitsutaka Takada

**Affiliations:** 1 Ebisu Pharmacy, 2-7-24, Motomachi, Naniwa-ku, Osaka-shi, Osaka, Japan; 2 Division of Clinical Drug Informatics, School of Pharmacy, Kindai University, 3-4-1, Kowakae, Higashi-osaka, Osaka, Japan

**Keywords:** adverse event, amiodarone, antidiabetic drug, Food and Drug Administration adverse event reporting system, metformin

## Abstract

**Background:** The association between metformin and amiodarone-induced adverse events was examined using spontaneous adverse event database. Additionally, the association between other antidiabetic drugs and amiodarone-induced adverse events were also examined.

**Methods:** A total of 6,153,696 reports from the first quarter of 2004 through the fourth quarter of 2015 were downloaded from the US Food and Drug Administration adverse event reporting system. Reporting odds ratio (ROR) and information component (IC) were used to detect associations between antidiabetic drugs and amiodarone-associated adverse events. Additionally, subset data analysis was performed to investigate whether the use of antidiabetic drugs further increased or decreased the risk of adverse events in patients receiving amiodarone therapy. Next, the RORs were adjusted for coadministered antidiabetic drugs using logistic regression analysis.

**Results:** By whole dataset analysis, significant inverse associations were found between metformin and interstitial lung disease (ROR 0.84, 95% confidence interval [CI] 0.79-0.90; IC -0.24, 95% CI -0.33 to -0.15). In the subset data analysis, metformin (ROR 0.62, 95%CI 0.43-0.89; IC -0.63, 95%CI -1.14 to -0.11), sulfonylureas (ROR 0.53, 95%CI 0.32-0.85; IC -0.85, 95%CI -1.53 to -0.17), and dipeptidyl peptidase-4 (DPP-4) inhibitors (ROR 0.25, 95%CI 0.08-0.78; IC -1.66, 95%CI -3.08 to -0.23) were inversely associated with hyperthyroidism. Additionally, metformin (ROR 0.43, 95%CI 0.33-0.57; IC -1.09, 95%CI -1.49 to -0.69), sulfonylureas (ROR 0.64, 95%CI 0.48-0.86; IC -0.59, 95%CI -1.00 to -0.17), and DPP-4 inhibitors (ROR 0.47, 95%CI 0.27-0.81; IC -0.99, 95%CI -1.76 to -0.22) were inversely associated with interstitial lung disease. In the logistic regression analyses, DPP-4 inhibitors (adjusted ROR 0.32, 95% CI 0.10-1.00) and metformin (adjusted ROR 0.46, 95% CI 0.34-0.62) were inversely associated with amiodarone-associated hyperthyroidism and interstitial lung disease, respectively.

**Conclusion:** Metformin is a candidate drug to reduce the risk of amiodarone-induced hyperthyroidism and interstitial lung disease.

## Introduction

Amiodarone, a class III antiarrhythmic drug that is used for the treatment of recurrent severe ventricular arrhythmia, paroxysmal atrial tachycardia, and atrial fibrillation as well as the maintenance of sinus rhythm after cardioversion of atrial fibrillation 1, is however associated with a number of significant adverse events 2, 3 including interstitial lung disease, hepatotoxicity, arrhythmia, thyrotoxicosis, and hypothyroidism 3-5. These adverse events may pose a serious threat to the patient, requiring the discontinuation of amiodarone. The underlying pathogenesis and definitive predictors of these adverse events remain unknown; however, several studies suggest that oxidative stress and mitochondrial dysfunction may play a role in amiodarone toxicity 6, 7. Conversely, metformin, a commonly prescribed biguanide antidiabetic drug to lower blood glucose in patients with type 2 diabetes, was recently demonstrated to suppress oxidative stress in cells of various organs and reduce markers of oxidative stress in patients with type 2 diabetes 8-11. Type 2 diabetes is the most common type of diabetes in adults and is often a comorbidity in patients with atrial fibrillation 12. Many patients with concomitant heart disease and type 2 diabetes may be treated with amiodarone and antidiabetic drugs in combination. However, no study to date elucidated the potential association between metformin and amiodarone-induced adverse events.

Spontaneous adverse event databases are utilized in signal detection for potential drug-associated adverse events. The US Food and Drug Administration (FDA) adverse event reporting system (FAERS) is a large and useful database for spontaneous adverse events, which can be utilized to identify unknown drug-associated adverse events using disproportionality analysis algorithms where reporting odds ratio (ROR) and information component (IC) are widely used parameters in detecting signals for drug-associated adverse events 13-18. Recently, Zhao *et al*. utilized a FAERS-based approach to identify a concomitantly used drug B that might mitigate the risk of adverse events associated with the use of drug A 18. Additionally, Nagashima *et al.* reported a FAERS-based approach to determine an effective drug combination that could lower known adverse events 19. Therefore, we utilized the FAERS to examine if metformin was associated with a decreased risk of amiodarone-induced adverse events. Furthermore, given that hyperglycemia can precipitate oxidative stress 20, we also examine the association between antidiabetic drugs and amiodarone-induced adverse events.

## Methods

### Materials

This retrospective study included data analysis of adverse event reports based on the FAERS, a computerized information database designed to support post-marketing safety surveillance programs for all approved drugs and therapeutic biological products. Adverse events and medication errors recorded in the FAERS database were downloaded from the FDA website (http://www.fda.gov/Drugs/InformationOnDrugs/ucm135151.htm). The system contains all reports of adverse events reported spontaneously by healthcare professionals, manufacturers, and consumers worldwide. The database comprises seven datasets: patient demographic and administrative information (file descriptor, DEMO), drug and biologic information (file descriptor, DRUG), adverse events (file descriptor, REAC), patient outcomes (file descriptor, OUTC), report sources (file descriptor, RPSR), start and end dates of drug therapy (file descriptor, THER), and indications for use/diagnosis (file descriptor, INDI). A relational database that concatenated the seven datasets by unique identification numbers for each FAERS report was built. The current study included data from the first quarter of 2004 through the fourth quarter of 2015. Reports with a common case number were identified as duplicate reports, and all duplicates were removed and excluded from all analyses. Finally, a total of 6,153,696 reports were included in the study.

### Identification of drugs

The FAERS permits the registration of arbitrary drug names including trade names, generic names, and abbreviations. All drug names were extracted from the DRUG file of the FAERS and recorded. A drug name archive that included the name of all preparations, generic names, and drug synonyms marketed around the globe was created using the Martindale website (https://www.medicinescomplete.com/mc/login.ht m). Although metformin is a main target drug in the current study, we included other antidiabetic drugs for comparison (Table 1). All records including antidiabetic drugs were selected, and relevant reactions were identified.

### Identification of adverse events

Adverse events analyzed in the current study were based on the definition provided by the Medical Dictionary for Regulatory Activities (MedDRA^®^ ver. 20,0; http://www.meddra.org/). Adverse events in the FAERS were coded using the MedDRA^®^ preferred terms (PTs), which are grouped by defined medical condition or area of interest. We identified PTs related to hyperthyroidism, hypothyroidism, interstitial lung disease, and hepatic disorder using the Standardised MedDRA^®^ Queries (SMQ). Hepatic disorder contains nine items of SMQ: drug related hepatic disorders - comprehensive search; cholestasis and jaundice of hepatic origin; drug related hepatic disorders - severe events only; hepatic failure, fibrosis and cirrhosis, and other liver damage-related conditions; hepatitis, non-infectious; liver neoplasms, benign (incl cysts and polyps); liver neoplasms, malignant and unspecified; liver-related investigations, signs and symptoms; and liver-related coagulation and bleeding disturbances. SMQs usually contain two PT categories corresponding to a “narrow” scope and a “broad” scope. The narrow and broad categories allow for the identification of cases that are highly likely to represent the condition of interest and those that would be useful when a user seeks to identify all possible cases, including some that may prove to be of little or no interest on closer inspection, respectively. In the current study, only PTs included in the narrow category were targeted.

### Disproportionality analysis

For signal detection using spontaneous reports, we calculated RORs and ICs using the case/non-case method 13, 15. Reports containing the event of interest were defined as cases, whereas the remaining reports were defined as non-cases. ROR and IC, both disproportionality measures, are distinct: ROR is a non-Bayesian, frequentist method, whereas IC is a Bayesian method. Both algorithms were used to calculate signal scores and assess whether a drug was associated significantly with an adverse event based on a two-by-two frequency table. For ROR, a signal is detected if the lower limit of the two-sided 95% confidence interval (CI) is >1 15. For IC, a signal is detected if the lower limit of the 95%CI is >0 13. For ROR, an inverse signal was defined if the upper limit of the 95%CI was <1. For IC, an inverse signal was defined if the upper limit of the 95%CI was <0. In the current study, both methods were used to detect signals, and adverse events were considered as drug-associated when the two indices met the criteria outlined above. To investigate the association of amiodarone and antidiabetic drug groups with adverse events, signal scores were calculated using the entire data. In addition, subset data analysis was performed to investigate whether antidiabetic drugs reduced the risk of extracardiac adverse events in patients receiving amiodarone therapy. Analysis of specific patient subsets with common risk factors can mitigate the effects of confounding factors 21, 22. Therefore, cases with amiodarone treatment were extracted from the database and defined as subset data to calculate the subset ROR and IC values. Next, the RORs were adjusted for coadministered antidiabetic drugs using logistic regression analysis. The logistic model included the terms for metformin, sulfonylureas, dipeptidyl peptidase-4 (DPP-4) inhibitors, meglitinides, thiazolidinediones, and α-glucosidase inhibitors. Data management and analyses were conducted using Visual Mining Studio software (version 8.0; Mathematical Systems, Inc) and JMP (version 13; SAS Institute).

## Results

### Whole dataset analyses

A total of 6,011, 11,286, 37,993, and 222,806 reports of hyperthyroidism, hypothyroidism, interstitial lung disease, and hepatic disorders, respectively, were identified in the FAERS. The results of disproportionality analyses were summarized in Table 2. Briefly, significant associations were identified between amiodarone and hyperthyroidism, hypothyroidism, interstitial lung disease, and hepatic disorders. Additionally, many significant associations were found between antidiabetic drugs and hyperthyroidism, hypothyroidism, interstitial lung disease, and hepatic disorders. Conversely, no significant associations were found between hyperthyroidism and metformin, DPP-4 inhibitors, meglitinides, thiazolidinediones, or α-glucosidase inhibitors or between hypothyroidism and α-glucosidase inhibitors. Of note, significant inverse associations were found between metformin and interstitial lung disease (ROR 0.84, 95%CI 0.79-0.90; IC -0.24, 95%CI -0.33 to -0.15).

### Subset data analyses

The subset data analyses were performed using 30,508 reports by restricting the whole dataset to patients receiving amiodarone. There were a total of 711, 568, 1,645, and 3,820 reports of hyperthyroidism, hypothyroidism, interstitial lung disease, and hepatic disorders, respectively. The results of the subset data analyses are presented in Table 3. Metformin (ROR 0.62, 95%CI 0.43-0.89; IC -0.63, 95%CI -1.14 to -0.11), sulfonylureas (ROR 0.53, 95%CI 0.32-0.85; IC -0.85, 95%CI -1.53 to -0.17), and DPP-4 inhibitors (ROR 0.25, 95%CI 0.08-0.78; IC -1.66, 95%CI -3.08 to -0.23) were inversely associated with amiodarone-associated hyperthyroidism. Additionally, metformin (ROR 0.43, 95%CI 0.33-0.57; IC -1.09, 95%CI -1.49 to -0.69), sulfonylureas (ROR 0.64, 95%CI 0.48-0.86; IC -0.59, 95%CI -1.00 to -0.17), and DPP-4 inhibitors (ROR 0.47, 95%CI 0.27-0.81; IC -0.99, 95%CI -1.76 to -0.22) were inversely associated with amiodarone-associated interstitial lung disease. Finally, no significant associations between any of the antidiabetic drug groups and hypothyroidism or hepatic disorders. The results of the logistic regression analyses are presented in Table 4. DPP-4 inhibitors (adjusted ROR 0.32, 95% CI 0.10-1.00) and metformin (adjusted ROR 0.46, 95% CI 0.34-0.62) were inversely associated with amiodarone-associated hyperthyroidism and interstitial lung disease, respectively. Thiazolidinediones were associated with amiodarone-associated hypothyroidism (adjusted ROR 2.30, 95% CI 1.16-4.55) and interstitial lung disease (adjusted ROR 1.74, 95% CI 1.05-2.88).

## Discussion

The current study utilizing the FAERS database revealed that amiodarone was significantly associated with increased risk of hyperthyroidism, hypothyroidism, interstitial lung disease, and hepatic disorders, which are serious amiodarone-induced adverse events that are well recognized in clinical practice, and indicated that the FAERS analysis was able to detect the risk of amiodarone-induced adverse events. In addition, our analyses also detected significant associations between certain antidiabetic drugs and adverse events (Table 2). However, diabetic patients have a high incidence of thyroid dysfunction and hepatic disorders 23, 24; thus, the association between antidiabetic drugs and thyroid dysfunction and hepatic disorders are uncertain. The associations between antidiabetic drugs and the adverse events identified in the current study might be attributable to confounding effects. Meanwhile, the association between diabetes mellitus and interstitial lung disease was unknown. Furthermore, the significant inverse association between metformin and interstitial lung disease, suggesting that metformin might mitigate the risk of interstitial lung disease, warrants further scrutiny. Studies previously reported that metformin attenuated gefitinib-induced and bleomycin-induced pulmonary fibrosis 11, 25-27. Furthermore, several studies reported that metformin attenuated radiation-induced pulmonary fibrosis 28. The current study results are therefore consistent with these previous reports showing that metformin attenuates the risk of pulmonary fibrosis.

We also examined whether metformin attenuated the risk of the amiodarone-associated adverse events using the subset data that were restricted to the patients who were treated with amiodarone and consequently identified a significant inverse association between metformin and interstitial lung disease, which implicated metformin as a candidate drug to attenuate the risk of amiodarone-induced interstitial lung disease. The inverse association between metformin and interstitial lung disease was also identified in the logistic regression analysis, which adjusted confounding factors including coadministered antidiabetic drugs. This result strongly suggested that metformin attenuates the risk of the amiodarone-induced interstitial lung disease. Amiodarone and its metabolites indirectly damage lung tissue by an immune response 29 and directly damage lung tissue by inducing the production of toxic O_2_ radicals which can damage cells directly 30. Sato *et al*. reported that metformin, an adenosine monophosphate-activated protein kinase activator, attenuated the development of lung fibrosis by inhibiting transforming growth factor signaling through the suppression of NADPH oxidase and reactive oxygen species 31. Additionally, metformin reduces mitochondrial reactive oxygen species 9. Therefore, metformin may reduce the risk of pulmonary toxicity caused by amiodarone-mediated accumulation of O_2_ radicals.

The current study also revealed that metformin was associated with a decreased risk of amiodarone-associated hyperthyroidism based on the analysis of the subset data restricted to the patients treated with amiodarone, suggesting that metformin attenuates the risk of amiodarone-induced hyperthyroidism. Meanwhile, the logistic regression analysis did not identify any inverse association between metformin and hyperthyroidism. Therefore, the association between metformin and hyperthyroidism remains unclear. Amiodarone-induced hyperthyroidism is primarily due to the direct effect of amiodarone on thyroid cells; however, excess iodide released from amiodarone may contribute to its toxicity 32. An experimental study suggested that amiodarone increased reactive oxygen species levels 6. When amiodarone-induced pro-oxidant activity exceeds the endogenous antioxidant capacity, thyroid follicles are destroyed, potentially resulting in amiodarone-induced thyrotoxicosis 7. Although there is no report on the antioxidant effect of metformin in thyroid cells, several studies reported on the antioxidant effect of metformin in other tissues, including umbilical vein endothelial cells and atrial myocytes 9, 33. Metformin inhibited the production of hyperglycemia-induced intracellular and mitochondrial reactive oxygen species and increased the expression of manganese superoxide dismutase in cultured human umbilical vein endothelial cells 9. Other studies reported that metformin attenuated tachycardia-induced oxidative stress and the subsequent adverse remodeling of atrial myocytes 33. The antioxidative activity of metformin might be associated with the reduction of cytotoxicity caused by amiodarone-mediated reactive oxygen species accumulation in the thyroid tissue. These various lines of evidence suggest metformin as a potential therapeutic to attenuate the risk of amiodarone-induced hyperthyroidism. Further studies are needed to clarify the association between metformin and hyperthyroidism.

The current study revealed that DPP-4 inhibitors are also associated with a decreased risk of amiodarone-induced hyperthyroidism in the analysis of the subset data and logistic regression analysis. Hyperglycemia is a major contributor to oxidative stress 34, 35. The normalization of blood glucose levels by antidiabetic medications may reduce oxidative stress and thereby be associated with a reduced risk of the development of amiodarone-associated hyperthyroidism. Therefore, antidiabetic therapy using antidiabetic drugs may attenuate the risk of amiodarone-induced hyperthyroidism. However, the logistic regression analyses identified a significant inverse association between DPP-4 inhibitors and hyperthyroidism but not between the other antidiabetic drugs and hyperthyroidism. This result implies DPP-4 inhibitors specifically attenuate the risk of amiodarone-induced hyperthyroidism. Some studies suggested that the administration of DPP-4 inhibitors relieves oxidative stress 36-39. Rizzo *et al.* reported that reduction in mean amplitude of glycemic excursions caused by vildagliptin and sitagliptin is associated with reduction of oxidative stress and markers of systemic inflammation in type 2 diabetic patients 39. Although there is no report on the antioxidant effect of DPP-4 inhibitors in thyroid cell, several studies reported on the antioxidant effect of DPP-4 inhibitors in blood plasma, heart, and kidney in rat and human umbilical vein endothelial cells and THP-1 36, 38. Further studies are needed to clarify the association between DPP-4 inhibitors and hyperthyroidism and elucidate the mechanism of the effect of DPP-4 inhibitors on hyperthyroidism.

There are several inherent limitations that should be taken into account when interpreting results obtained from the FAERS database. First, not all adverse events observed in clinical settings are included in the database. Second, the FAERS database contains missing data, misspelled drug names or duplicate data. To overcome problems with data quality, we had deleted or corrected such data before conducting analysis in this study. Third, several variables were also limited in our FAERS analysis; age, sex, race, treatment duration, or drug dosage were not considered. Finally, there were some separations between the results of the subset analysis and the adjusted ROR. Various methods have been reported for detecting the signals related to the drug interaction 40, 41. However, the difference in method may have an effect on the results, and it is unclear which result is more correct. It will be necessary to conduct further detailed verification using these methods in the future.

The current study results provide evidence for metformin as a candidate drug that might attenuate the risk of hyperthyroidism and interstitial lung disease in patients treated with amiodarone. Furthermore, for interstitial lung disease, the potential beneficial effect of metformin may not be limited only to patients treated with amiodarone. As signal detection using the FAERS should not be interpreted as assuming a causal relation between drugs and clinical events, the hypotheses generated by the FAERS needs to be validated using other methods. Therefore, experimental and clinical studies are warranted to elucidate the effect of metformin on amiodarone-induced interstitial lung disease and hyperthyroidism.

## Conclusion

The current study based on the analysis of the FAERS database revealed that metformin was associated with a decreased risk of amiodarone-associated interstitial lung disease and hyperthyroidism in patients treated with amiodarone. Furthermore, the results indicated that metformin was associated with a decreased risk of interstitial lung disease in the whole dataset analysis. Overall, these findings raise the possibility of metformin as a therapeutic option to attenuate the risk of hyperthyroidism and interstitial lung disease in patients treated with amiodarone. Furthermore, for interstitial lung disease, this potentially beneficial effect of metformin may not be limited only to patients treated with amiodarone.

## Figures and Tables

**Table 1:** Oral antidiabetic drugs

Sulfonylureas	Chlorpropamide
	Acetohexamide
	Glyclopyramide
	Glibenclamide
	Gliclazide
	Glimepiride
	Tolbutamide
**Biguanides**	Metformin
**Thiazolidinediones**	Pioglitazone
**α-Glucosidase inhibitors**	Acarbose
	Voglibose
	Miglitol
**Meglitinides**	Nateglinide
	Mitiglinide
	Repaglinide
**DPP-4 inhibitors**	Sitagliptin
	Vildagliptin
	Alogliptin
	Linagliptin
	Teneligliptin
	Anagliptin
	Saxagliptin
	Trelagliptin
	Omarigliptin

DPP-4: dipeptidyl peptidase 4

**Table 2:** The associations between amiodarone and specific antidiabetic drug groups and adverse events

	Case	Non-cases	ROR	95%CI	IC	95%CI	Signal
**Hyperthyroidism**							
Amiodarone	711	29,797	27.54	25.45-29.81	4.53	4.42-4.64	↑
Metformin	181	190,090	0.97	0.84-1.13	-0.04	-0.25-0.17	nd
Sulfonylureas	97	78,250	1.27	1.04-1.56	0.34	0.05-0.63	↑
DPP-4 inhibitors	44	55,027	0.82	0.61-1.10	-0.28	-0.71-0.14	nd
Meglitinides	12	9,852	1.25	0.71 -2.20	0.29	-0.50-1.07	nd
Thiazolidinediones	40	33,984	1.21	0.88-1.64	0.26	-0.18-0.70	nd
α-Glucosidase inhibitors	8	7,407	1.10	0.55-2.21	0.13	-0.82-1.07	nd
**Hypothyroidism**							
Amiodarone	568	29,940	10.82	9.94-11.78	3.32	3.20-3.44	↑
Metformin	484	189,787	1.41	1.28-1.54	0.47	0.34-0.60	↑
Sulfonylureas	236	78,111	1.66	1.46-1.89	0.71	0.53-0.90	↑
DPP-4 inhibitors	170	54,901	1.70	1.46-1.97	0.75	0.53-0.96	↑
Meglitinides	33	9,831	1.83	1.30-2.58	0.83	0.35-1.32	↑
Thiazolidinediones	92	33,932	1.48	1.20-1.82	0.55	0.26-0.85	↑
α-Glucosidase inhibitors	15	7,400	1.10	0.66-1.83	0.13	-0.58-0.84	nd
**Interstitial lung disease**							
Amiodarone	1,645	28,863	9.54	9.07-10.04	3.12	3.05-3.19	↑
Metformin	997	189,274	0.84	0.79-0.90	-0.24	-0.33 to -0.15	↓
Sulfonylureas	850	77,497	1.78	1.67-1.91	0.81	0.71-0.91	↑
DPP-4 inhibitors	476	54,595	1.41	1.29-1.54	0.48	0.35-0.61	↑
Meglitinides	148	9,716	2.46	2.09-2.89	1.27	1.03-1.50	↑
Thiazolidinediones	263	33,761	1.26	1.11-1.42	0.32	0.15-0.50	↑
α-Glucosidase inhibitors	206	7,209	4.62	4.02-5.31	2.15	1.95-2.35	↑
**Hepatic disorder**							
Amiodarone	3,820	26,688	3.86	3.73-3.99	1.79	1.74-1.84	↑
Metformin	8,389	181,882	1.24	1.21-1.26	0.28	0.25-0.32	↑
Sulfonylureas	4,726	73,621	1.72	1.67-1.78	0.74	0.69-0.78	↑
DPP-4 inhibitors	2,830	52,241	1.45	1.39-1.50	0.50	0.45-0.56	↑
Meglitinides	806	9,058	2.37	2.21-2.55	1.17	1.07-1.28	↑
Thiazolidinediones	1,322	32,702	1.08	1.02-1.14	0.10	0.02-0.18	↑
α-Glucosidase inhibitors	1,035	6,380	4.33	4.06-4.63	1.94	1.85-2.04	↑

CI: confidence interval; DPP-4: dipeptidyl peptidase 4; IC: information component; ROR: reporting odds ratio. nd: no signal detected; ↓: negative signal; ↑: positive signal.

**Table 3:** The associations between amiodarone-associated adverse events and antidiabetic drug groups

	Case	Non-cases	ROR	95%CI	IC	95%CI	Signal
**Hyperthyroidism**							
Metformin	31	2,043	0.62	0.43-0.89	-0.63	-1.14 to -0.11	↓
Sulfonylureas	17	1,327	0.53	0.32-0.85	-0.85	-1.53 to -0.17	↓
DPP-4 inhibitors	3	494	0.25	0.08-0.78	-1.66	-3.08 to -0.23	↓
Meglitinides	4	269	0.62	0.23-1.67	-0.56	-1.84-0.72	nd
Thiazolidinediones	6	226	1.11	0.49-2.51	0.12	-0.97-1.21	nd
α-Glucosidase inhibitors	0	123	-	-	-	-	-
**Hypothyroidism**							
Metformin	29	2,045	0.73	0.50-1.07	-0.40	-0.94-0.13	nd
Sulfonylureas	28	1,316	1.13	0.77-1.66	0.15	-0.39-0.70	nd
DPP-4 inhibitors	7	490	0.75	0.35-1.59	-0.36	-1.38- 0.65	nd
Meglitinides	3	270	0.58	0.19-1.83	-0.61	-2.04-0.82	nd
Thiazolidinediones	8	224	1.90	0.93-3.86	0.75	-0.22-1.72	nd
α-Glucosidase inhibitors	0	123	-	-	-	-	-
**Interstitial lung disease**							
Metformin	52	2,022	0.43	0.33-0.57	-1.09	-1.49 to -0.69	↓
Sulfonylureas	48	1,296	0.64	0.48-0.86	-0.59	-1.00 to -0.17	↓
DPP-4 inhibitors	13	484	0.47	0.27-0.81	-0.99	-1.76 to -0.22	↓
Meglitinides	15	258	1.02	0.60-1.72	0.02	-0.71-0.75	nd
Thiazolidinediones	16	216	1.30	0.78-2.17	0.33	-0.39-1.04	nd
α-Glucosidase inhibitors	7	116	1.06	0.49-2.27	0.06	-0.98-1.09	nd
**Hepatic disorder**							
Metformin	236	1,838	0.89	0.77-1.02	-0.14	-0.34-0.06	nd
Sulfonylureas	185	1,159	1.12	0.96-1.31	0.13	-0.09-0.36	nd
DPP-4 inhibitors	55	442	0.87	0.65-1.15	-0.18	-0.58-0.22	nd
Meglitinides	38	235	1.13	0.80-1.60	0.14	-0.34-0.63	nd
Thiazolidinediones	31	201	1.08	0.74-1.58	0.08	-0.45-0.62	nd
α-Glucosidase inhibitors	23	100	1.61	1.02-2.54	0.54	-0.09-1.17	nd

CI: confidence interval; DPP-4: dipeptidyl peptidase 4; IC: information component; ROR: reporting odds ratio. nd: no signal detected; ↓: negative signal; ↑: positive signal.

**Table 4:** Adjusted ROR of antidiabetic drugs for amiodarone- associated extracardiac adverse events in subset data

	Adjusted ROR	95% CI	Signal
**Hyperthyroidism**			
Metformin	0.74	0.51-1.07	nd
Sulfonylureas	0.61	0.37-1.01	nd
DPP-4 inhibitors	0.32	0.10-1.00	↓
Meglitinides	0.71	0.26-1.92	nd
Thiazolidinediones	1.41	0.62-3.21	nd
α-Glucosidase inhibitors	-	-	-
**Hypothyroidism**			
Metformin	0.72	0.48-1.06	nd
Sulfonylureas	1.22	0.82-1.84	nd
DPP-4 inhibitors	0.79	0.37-1.71	nd
Meglitinides	0.59	0.19-1.87	nd
Thiazolidinediones	2.30	1.16-4.55	↑
α-Glucosidase inhibitors	-	-	-
**Interstitial lung disease**			
Metformin	0.46	0.34-0.62	↓
Sulfonylureas	0.80	0.59-1.09	nd
DPP-4 inhibitors	0.62	0.35-1.09	nd
Meglitinides	1.17	0.69-1.98	nd
Thiazolidinediones	1.74	1.05-2.88	↑
α-Glucosidase inhibitors	1.18	0.54-2.57	nd
**Hepatic disorder**			
Metformin	0.87	0.75-1.00	nd
Sulfonylureas	1.18	0.99-1.39	nd
DPP-4 inhibitors	0.87	0.65-1.17	nd
Meglitinides	1.15	0.81-1.63	nd
Thiazolidinediones	1.13	0.78-1.64	nd
α-Glucosidase inhibitors	1.52	0.96-2.41	nd

CI: confidence interval; DPP-4: dipeptidyl peptidase 4; ROR: reporting odds ratio. nd: no signal detected; ↓: negative signal; ↑: positive signal.
